# Older Adults’ Attitude Towards and Intent to Use of AI-Powered Conversational Agents for Social Support

**DOI:** 10.1093/geroni/igaf122.1329

**Published:** 2025-12-31

**Authors:** Ka Lon Sou, Matthew Zhi Yuan Lau, Fuxi Ouyang, W Quin Yow

**Affiliations:** Singapore University of Technology and Design, Singapore, Singapore; Singapore University of Technology and Design, Singapore, Singapore; Singapore University of Technology and Design, Singapore, Singapore; Singapore University of Technology and Design, Singapore, Singapore

## Abstract

Social isolation affects nearly one-fourth of those aged 65+ in the U.S., and imposes risks to one’s health. Recent advancement of large language model (LLM) suggests that artificial intelligence (AI)-powered chatbot may foster social support. Our lab, thus, developed an age-friendly LLM-powered chatbot, Ami Chat, that aims to provide social support for (isolated) older adults. In this study, we aim to identify the attitude-related factors and AI-related characteristics that influence older adults’ intent to use an AI-powered chatbot. Thirty-two participants (21 females, Mage=72.28, range=66-85) interacted with Ami Chat for around 20 minutes, and then completed questionnaires that evaluated their attitudes towards Ami Chat, their perception on Ami Chat’ AI-related characteristics, as well as their intent to use of Ami Chat in their daily lives. Our results showed that the AI social interaction quality (AISI) was significantly predicting intent to use of the chatbot (β = 1.30, p<.001). AI anthropomorphism was negatively predicting intent to use (β=-0.65, p=.004). Health (β = 0.48, p=.043), sociability (β = 0.81, p=.007), agency (β=-0.78, p=.025) and perceived empathy (β = 0.76, p=.004) significantly predicted AISI. Attitude towards Ami Chat was only significantly predicted by perceived empathy (β = 1.39, p=.0032). Our results highlight the importance of social interaction closeness, sociability, agency, and perceived empathy in shaping older adults’ attitude towards and intent to use AI-powered chatbots in their daily lives. Notably, our results also suggest that overly human-like AI may not always appeal to older users. These insights emphasize that there are age-specific needs in the design of the AI-driven solutions for social support.

